# Oxamate Improves Glycemic Control and Insulin Sensitivity via Inhibition of Tissue Lactate Production in *db/db* Mice

**DOI:** 10.1371/journal.pone.0150303

**Published:** 2016-03-03

**Authors:** Weiran Ye, Yijia Zheng, Shanshan Zhang, Li Yan, Hua Cheng, Muchao Wu

**Affiliations:** Department of Endocrinology, Sun Yat-sen Memorial Hospital, Sun Yat-sen University, Guangzhou, P. R. China; GDC, GERMANY

## Abstract

Oxamate (OXA) is a pyruvate analogue that directly inhibits the lactate dehydrogenase (LDH)-catalyzed conversion process of pyruvate into lactate. Earlier and recent studies have shown elevated blood lactate levels among insulin-resistant and type 2 diabetes subjects and that blood lactate levels independently predicted the development of incident diabetes. To explore the potential of OXA in the treatment of diabetes, *db/db* mice were treated with OXA in vivo. Treatment of OXA (350–750 mg/kg of body weight) for 12 weeks was shown to decrease body weight gain and blood glucose and HbA1c levels and improve insulin secretion, the morphology of pancreatic islets, and insulin sensitivity in *db/db* mice. Meanwhile, OXA reduced the lactate production of adipose tissue and skeletal muscle and serum lactate levels and decreased serum levels of TG, FFA, CRP, IL-6, and TNF-α in *db/db* mice. The PCR array showed that OXA downregulated the expression of Tnf, Il6, leptin, Cxcr3, Map2k1, and Ikbkb, and upregulated the expression of Irs2, Nfkbia, and Pde3b in the skeletal muscle of *db/db* mice. Interestingly, LDH-A expression increased in the islet cells of *db/db* mice, and both treatment of OXA and pioglitazone decreased LDH-A expression, which might be related to the improvement of insulin secretion. Taken together, increased lactate production of adipose tissue and skeletal muscle may be at least partially responsible for insulin resistance and diabetes in *db/db* mice. OXA improved glycemic control and insulin sensitivity in *db/db* mice primarily via inhibition of tissue lactate production. Oxamic acid derivatives may be a potential drug for the treatment of type 2 diabetes.

## Introduction

Oxamate (OXA) is a pyruvate analogue that directly inhibits the conversion of pyruvate into lactate by lactate dehydrogenase (LDH) [1]. OXA has been used for the research of physiological and pathogenic role of LDH in vitro or in animals in vivo. For example, most cancer cells rely on aerobic glycolysis instead of oxidative phosphorylation to generate metabolic energy, a phenomenon called the Warburg effect [2]. Aerobic glycolysis is a hallmark of malignant tumor metabolism, which is associated with the progression of tumors [3, 4]. It has been found that OXA inhibits the activity of LDH and decreases lactate production in cancer cells [5, 6]. Oxamic acid derivatives are considered potential drugs for the treatment of malignant tumors [7].

Earlier work suggests that blood lactate is elevated among obese, insulin-resistant subjects, and that blood lactate may be an independent risk factor for the development of type 2 diabetes [8–11]. Elevated lactate may promote hepatic gluconeogenesis and interfere with glucose uptake in the muscle by serving as a substitute for glucose utilization [12]. Lactate infusion also decreases glucose oxidation [13]. In 2010, a large sample retrospective study by Crawford and colleagues showed that plasma lactate was strongly associated with type 2 diabetes [14]. In 2013, a prospective study by Juraschek and colleagues reported that blood lactate predicted incident diabetes independent of many other risk factors and was strongly related to markers of insulin resistance [15].

In recent years, some researchers put forward the concept of ‘disallowed beta-cell genes’ [16, 17]. Disallowed beta-cell genes are a group of genes whose expression is specifically inhibited in beta cells to avoid inappropriate release of insulin [16]. Recent work has identified over 60 genes that are specifically repressed in beta cells. LDH-A is one of the ‘founder’ members of this group of genes [16]. In vivo and in vitro studies have shown that high glucose levels upregulate the expression of LDH-A mRNA in rodent and human pancreatic islets [18–20]. Increased expression of LDH-A may impair insulin secretion in beta cells [21, 22].

Increased FFA load can cause multiple dysregulations, which are collectively known as lipotoxicity [23]. Lipotoxicity constitutes an important pathogenic link between obesity, insulin resistance, and type 2 diabetes [24]. On the other hand, type 2 diabetes and obesity are characterized by low-grade, chronic inflammation. [25]. Pro-inflammatory cytokines might predict the development of type 2 diabetes [26–28]; inflammation increases insulin resistance, which leads to type 2 diabetes [29]. It was observed that lactate boosted the gene transcription of pro-inflammatory cytokines in macrophages, and OXA inhibited lactate output and FFA synthesis in 3T3-L1 cells, and improved insulin resistance induced by palmitate in myotubes [30–32].

Although the above studies indicate that OXA may have anti-diabetic properties, the effects of OXA in vivo in diabetic animal models have not yet been investigated. In the present study, we investigated the effects of OXA treatment on the glycemic control, insulin resistance, and the associated mechanisms in vivo in *db/db* mice, to explore the possibility of oxamic acid derivatives being used as potential drugs for the treatment of type 2 diabetes.

## Materials and Methods

### Animals

The *db/db* (BKS.Cg-Dock7m+/+Leprdb/JNju) mice and *db/+* ([Leprdb]mut/wt) mice were purchased from Model Animal Research Center of Nanjing University (Nanjing, China). This study was carried out in strict accordance with the recommendations in the guide for the care and use of animals of the Association for the Committee for Animal Experiments of National Center. The protocol was approved by the Ethics Committee of Sun Yat-sen University. The mice were housed individually with free access to food and water on a 12 hour light/dark cycle.

### Study design

Thirty-five four-week-old male mice were randomly divided into 7 groups (numbered as groups I–VII) with five mice in each group. Group I were *db/+* mice treated with normal saline (NS, 0.9% NaCl) by intraperitoneal injection (vehicle *db/+* group), group II–VII were *db/db* mice administrated with NS (vehicle *db/db* group), 150, 350, 550, and 750 mg/kg of body weight of OXA (Sigma-Aldrich, St. Louis, MO, USA) by intraperitoneal injection, and 100 mg/kg of body weight of pioglitazone (PIO, Takeda Pharmaceutical, Osaka, Japan) by oral gavage, respectively, daily for 12 weeks. The condition (including activity, posture, pelage, and skin) of the mice was observed daily. Food intake (estimated by collecting and weighing uneaten food) was determined three times per week. Fasting blood glucose (FBG) levels and body weight after 8-h fasting were monitored weekly. After treatment of 12 weeks, an intraperitoneal glucose tolerance test (IPGTT) and an insulin tolerance test (ITT) were performed. Then, after 8-h fasting, the mice were anesthetized with pentobarbital sodium (50 mg/kg, Sigma-Aldrich, St. Louis, MO, USA), blood samples were taken by cardiac puncture, and serum was separated and stored at -20°C for detection of lactate, glycosylated hemoglobin (HbA1c), triglyceride (TG), free fatty acid (FFA), C-reactive protein (CRP), interleukin-6 (IL-6), and tumor necrosis factor-α (TNF-α). The mice were then immediately sacrificed by cervical dislocation and adipose, skeletal muscle, and pancreas tissues were rapidly dissected. Adipose tissue and skeletal muscle were frozen in liquid nitrogen and stored at -70°C for the detection of lactate production and analysis of the PCR array, and the pancreas was fixed in 10% (v/v) formalin for the detection of insulin expression. The dosage of OXA and PIO was adjusted weekly according to body weight to maintain a similar dose per kilogram over the entire experiment.

In addition, after 8-h fasting, three 4-week-old male *db/+* mice were anesthetized with pentobarbital sodium (50 mg/kg), sacrificed by cervical dislocation, and the liver, adipose, skeletal muscle, and pancreas tissue were rapidly dissected and fixed in 10% (v/v) formalin. These samples and the pancreas sample from the above experimental mice were tested for LDH-A expression.

### Health score

Six criteria including activity, posture, dehydration, pelage, and skin wounding were used to assess the health of the mice (Table 1). The health score was determined daily throughout the study and scoring was performed in a blinded manner by two independent observers. The overall health score ranged from 0–10, and a health score of 0 indicated healthy, while mice with scores from 8 to 10 were considered euthanized according to our IACUC protocol.

**Table 1:** Health Score Criteria.

Criteria		Score
**Activity**	Active	0
	Moves slowly or moves only when stimulated	1
	Stationary	2
**Posture**	Normal	0
	Hunched and able to rear up	1
	Hunched and unable to rear up	2
**Dehydration**	Skin does not tent when picked up	0
	Skin tents but is able to return to the original shape	1
	Skin tents and stays up in a tent	2
**Pelage**	Smooth	0
	Partly ruffled	1
	All ruffled	2
**Skin wound**	Absent	0
	Present	1
	Present with purulent discharge	2

To evaluate the overall health condition, the activity, posture, pelage, and skin of the mice were observed and scoring was performed daily throughout the study. The overall health score ranged from 0–10, and a health score of 0 indicated healthy, while a score of 8 to 10 was considered euthanized.

### Intraperitoneal glucose tolerance test (IPGTT)

The IPGTT was carried out after an overnight 10 h fast by intraperitoneal injection of 2 g/kg of body weight of glucose. Blood samples were collected from tail tip vein at 0, 30, 60, and 120 min after glucose injection for the immediate detection of glucose, then blood samples were separated and serum was stored at -20°C for the detection of insulin. For insulin secretion, the percentage of insulin versus the 0 min time point was calculated.

### Insulin tolerance test (ITT)

An ITT was conducted after an overnight 10 h fast by intraperitoneal injection of 1 U/kg of body weight of insulin (Novolin R, Novo Nordisk, Copenhagen, Denmark). Blood samples were collected from the tail tip vein at 0, 15, 30, and 60 min after insulin injection for the detection of glucose. The percentage of blood glucose versus the 0 min time point was calculated.

### Blood biochemical marker measurements

Blood glucose concentrations were determined using a glucometer (LifeScan Inc., CA, USA). Serum insulin (Millipore, MO, USA), HbA1c (BlueGene Biotech, Shanghai, China), TG (BlueGene Biotech, Shanghai, China), CRP (R&D Systems, MN, USA), IL-6 (Neobioscience, Shenzhen, China), and TNF-α (Neobioscience, Shenzhen, China) levels were measured by enzyme-linked immunosorbent assay (ELISA). Serum lactate (BioVision, CA, USA) and FFA (BioVision, CA, USA) levels were assayed by a colorimetric method.

### Measurements of tissue lactate production

Adipose tissue and skeletal muscle samples were rapidly thawed, blotted dry, and weighed. A 0.1 g sample of adipose tissue and skeletal muscle were homogenized respectively in the buffer that contained 50 mmol/L of potassium phosphate, 1 mmol/L of EDTA, 1 mmol/L of DTT, and 0.05% TritonX-100 (pH 7.4). The homogenates were then centrifuged at 10,000 rpm for 15 min at 4°C, and the supernatants were collected for the detection of lactate production. The lactate (BioVision, CA, USA) production of adipose tissue and skeletal muscle were examined by a colorimetric method.

### PCR array analysis

Total RNA from the skeletal muscle of vehicle *db/db* mice (n = 3) and OXA (750 mg/kg of body weight)-treated mice (n = 3) was extracted using the TRIzol reagent (Invitrogen, CA, USA). RNA yield and quality were assessed by UV absorbance and agarose gel electrophoresis. Then, RNA was reverse transcribed to cDNA. Real-Time PCR array (PAMM-1562, SuperArray Bioscience Corp., MD, USA) was performed to scan for the expression of 84 genes related to the mouse insulin signaling pathway, according to manufacturer’s instructions. In brief, diluted cDNA was individually placed into the wells of the PCR array. Then Real-Time PCR was performed, and the thermal cycling conditions were as follows: initial polymerase activation/denaturation at 95°C for 10 min, followed by 40 cycles at 95°C for 15 sec, and 60°C for 1 min. The PCR array data were normalized by the housekeeping gene (β-actin) and expressed as the mean fold change in OXA-treated samples relative to the vehicle-treated samples.

### Immunohistochemical staining

Liver, adipose, skeletal muscle, and pancreas tissues were fixed in formalin, embedded in paraffin, and sectioned (4 μm). Paraffin sections were deparaffinized with xylene and hydrated with a series of graded ethanol washes. For insulin immunochemical analysis of the pancreas, the sections were incubated overnight at 4°C with insulin Rabbit Polyclonal Antibody (1:500, Santa Cruz Biotechnology, Inc., CA, USA). For LDH-A immunochemical analysis of liver, adipose, skeletal muscle, and pancreas tissue, the sections were incubated overnight at 4°C with LDH-A Rabbit Polyclonal Antibody (1:300, LifeSpan Biosciences, WA, USA). The sections were then washed, and incubated with anti-rabbit Plus-HRP (Dako Cytomation, Glostrup, Denmark) for 30 min at room temperature. The sections were then incubated with DAB and counterstained with hematoxylin.

### Statistical analysis

The data are presented as the mean ± standard deviation, and were analyzed using SPSS 13.0 statistical software. The Student’s t-test was used to compare two independent variables, and one way analysis of variance (ANOVA) followed by the Student-Newman-Keuls test was used to compare more than 2 variables. A *P* value of less than 0.05 was considered statistically significant.

## Results

### Health condition and food intake

**Fig 1:**
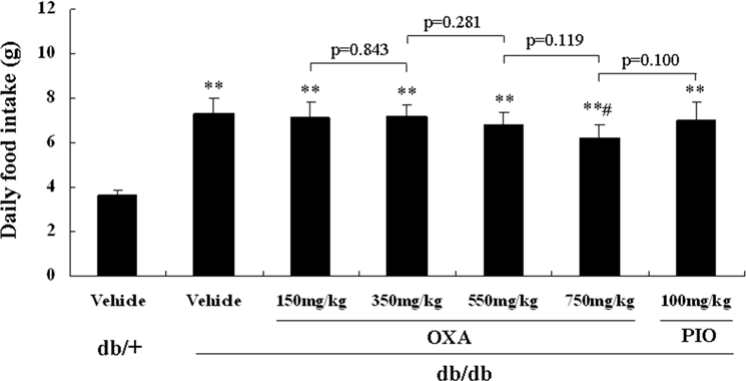
Effects of OXA on food intake. *db/+* mice received the vehicle (0.9% NaCl), and *db/db* mice received the vehicle and OXA (150, 350, 550, and 750 mg/kg of body weight) or PIO (100 mg/kg of body weight) for 12 weeks. Food intake (estimated by collecting and weighing uneaten food) was determined three times per week during the experimental period. Results are expressed as the mean ± SD, n = 5. OXA: oxamate; PIO: pioglitazone. **: *p* < 0.01 as compared to the vehicle *db/+* mice; #: *p* < 0.05 as compared to the vehicle *db/db* mice (one-way ANOVA followed by Student-Newman-Keuls test).

All of the mice were healthy (a health score of 0) throughout the study. Treatment with 150, 350, and 550 mg/kg of body weight of OXA or 100 mg/kg of body weight of PIO had no effect on food intake, while treatment with 750 mg/kg of body weight of OXA lightly decreased food intake in the *db/db* mice during the 12-week treatment (Fig 1).

### Effects of OXA on weight gain

**Fig 2:**
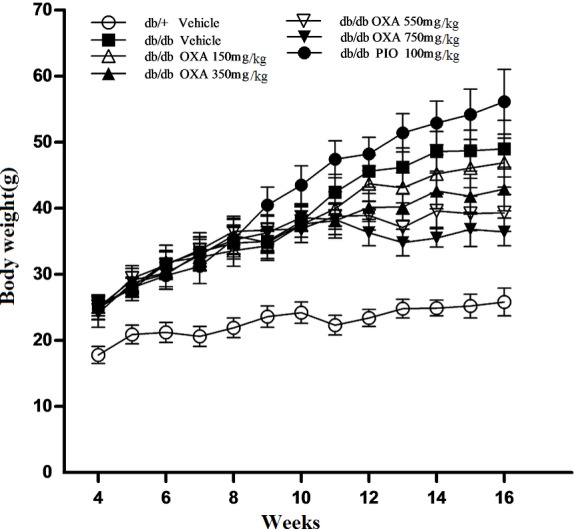
Effects of OXA on body weight gain. *db/+* mice received the vehicle (0.9% NaCl), and *db/db* mice received the vehicle and OXA (150, 350, 550, 750 mg/kg of body weight) or PIO (100 mg/kg of body weight) respectively for 12 weeks. Body weight after 8-h fasting was monitored weekly during the experiment period. Results are expressed as the mean ± SD, n = 5. OXA: oxamate; PIO: pioglitazone.

Changes in the body weight of all the studied mice during the 12 week period are shown in Fig 2. Although 150 mg/kg of body weight of OXA had not effect on inhibiting weight gain, from 11 weeks of age, the body weight of the *db/db* mice receiving OXA at 350, 550, and 750 mg/kg of body weight began to decrease compared with that of the vehicle *db/db* mice, and decreased by 12.4, 19.8, and 25.7%, respectively, at the age of 16 weeks (after treatment for 12 weeks). In contrast, from 9 weeks of age (except at 12 weeks of age) the body weight of the PIO-treated *db/db* mice began to increase compared with that of the vehicle *db/db* mice, and increased by 14.5% at the age of 16 weeks.

### Effects of OXA on glycemic control

**Fig 3:**
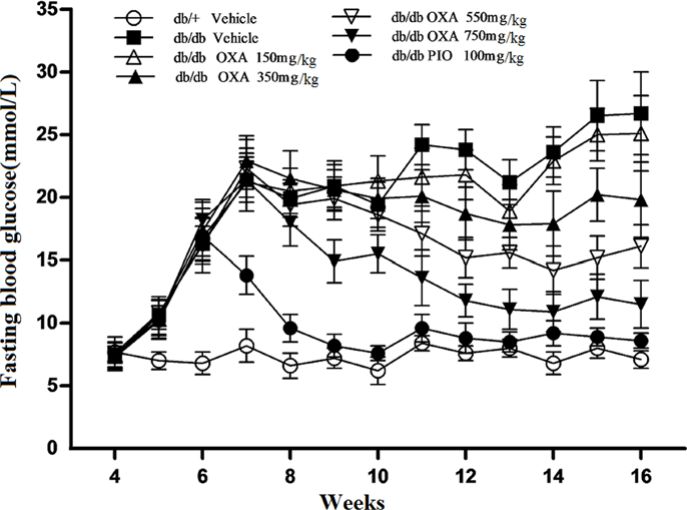
Effects of OXA on fasting blood glucose. *db/+* mice received the vehicle (0.9% NaCl), and *db/db* mice received the vehicle and OXA (150, 350, 550, 750 mg/kg of body weight) or PIO (100 mg/kg of body weight) respectively for 12 weeks. Blood glucose after 8-h fasting was monitored weekly during the experiment period. Results are expressed as the mean ± SD, n = 5. OXA: oxamate; PIO: pioglitazone.

**Fig 4:**
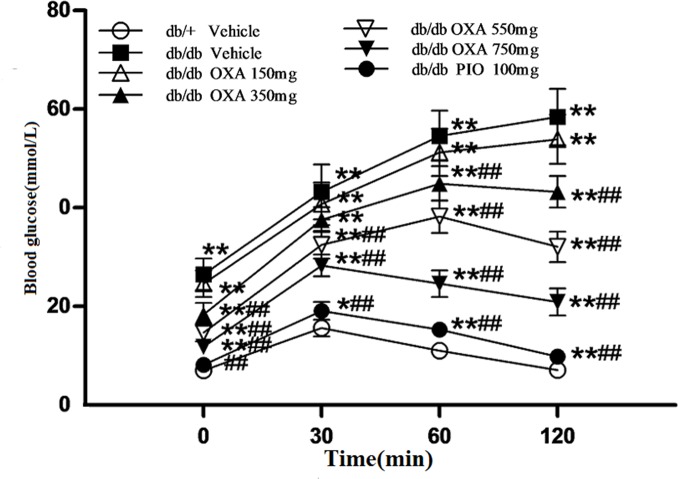
Effects of OXA on blood glucose levels during IPGTT. *db/+* mice received the vehicle (0.9% NaCl), and *db/db* mice received the vehicle and OXA (150, 350, 550, 750 mg/kg of body weight) or PIO (100 mg/kg of body weight) respectively for 12 weeks. After fasting for 10 h, the mice were subjected to a glucose tolerance test (GTT) by the intraperitoneal injection of 2 g/kg body weight of glucose. Blood was sampled and examined at 0 (baseline), 30, 60, and 120 min for glucose levels. Results are expressed as the mean ± SD, n = 5. OXA: oxamate; PIO: pioglitazone. *: *p* < 0.05 as compared to the vehicle *db/+* mice; **: *p* < 0.01 as compared to the vehicle *db/+* mice; ##: *p* < 0.01 as compared to the vehicle *db/db* mice (one-way ANOVA followed by Student-Newman-Keuls test).

**Fig 5:**
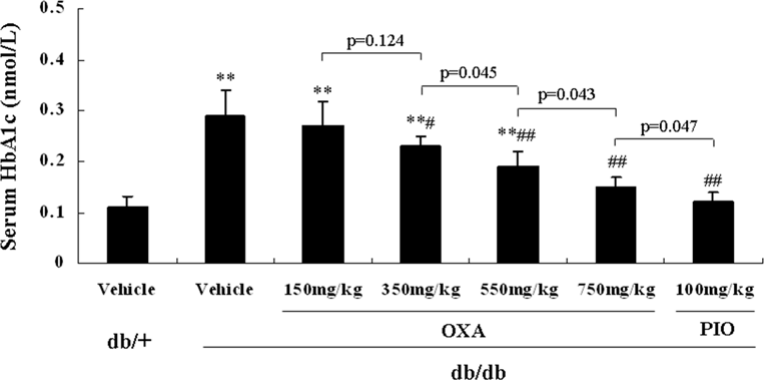
Effects of OXA on serum HbA1c levels. *db/+* mice received the vehicle (0.9% NaCl), and *db/db* mice received the vehicle and OXA (150, 350, 550, 750 mg/kg of body weight) or PIO (100 mg/kg of body weight) respectively for 12 weeks. Then, an 8-h fasting blood sample was collected for the detection of HbA1c levels. Results are expressed as the mean ± SD, n = 5. OXA: oxamate; PIO: pioglitazone. **: *p* < 0.01 as compared to the vehicle *db/+* mice; #: *p* < 0.05 as compared to the vehicle *db/db* mice; ##: *p* < 0.01 as compared to the vehicle *db/db* mice (one-way ANOVA followed by Student-Newman-Keuls test).

We found that there were no significant differences in glycemic control between the vehicle *db/db* mice and the *db/db* mice treated with 150 mg/kg of body weight of OXA. However, from 11 and 9 weeks of age, FBG levels in the *db/db* mice receiving OXA at 350, 550, and 750 mg/kg of body weight were lower than that in the vehicle *db/db* mice, and at the age of 16 weeks (after treatment for 12 weeks), the FBG levels were decreased by 25.8, 39.7, and 56.9% respectively (Fig 3). With the increase in the dose of OXA from 350 to 750 mg/kg of body weight, glucose tolerance improved gradually and HbA1c levels became lower in the *db/db* mice at the age of 16 weeks (Figs 4 and 5).

### Effects of OXA on insulin sensitivity

**Fig 6:**
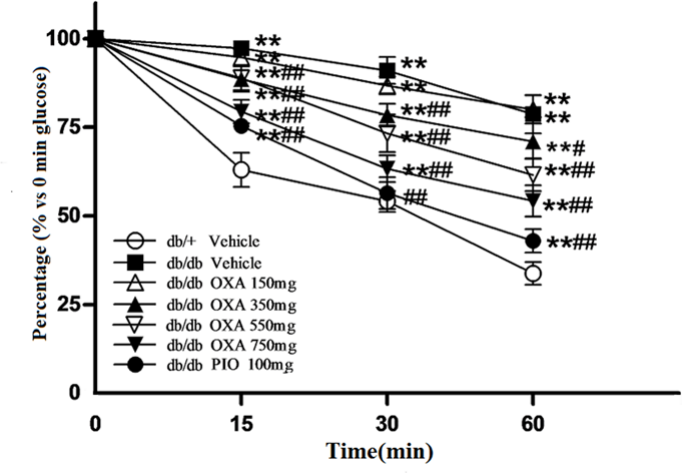
Effects of OXA on blood glucose levels during ITT. *db/+* mice received the vehicle (0.9% NaCl), and *db/db* mice received the vehicle and OXA (150, 350, 550, 750 mg/kg of body weight) or PIO (100 mg/kg of body weight) respectively for 12 weeks. After fasting for 10 h, the mice were subjected to an insulin tolerance test (ITT) by the intraperitoneal injection of 1 U/kg of body weight of insulin. Blood was sampled and examined at 0 (baseline), 15, 30, and 60 min for glucose. Results are expressed as the percentage (% versus 0 min glucose) ± SD, n = 5. OXA: oxamate; PIO: pioglitazone. **: *p* < 0.01 as compared to the vehicle *db/+* mice; #: *p* < 0.05 as compared to the vehicle *db/db* mice; ##: *p* < 0.01 as compared to the vehicle *db/db* mice (one-way ANOVA followed by Student-Newman-Keuls test).

**Fig 7:**
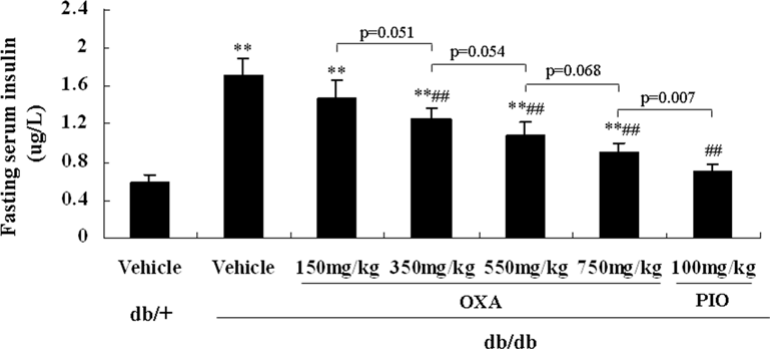
Effects of OXA on fasting serum insulin levels. *db/+* mice received the vehicle (0.9% NaCl), and *db/db* mice received the vehicle and OXA (150, 350, 550, 750 mg/kg of body weight) or PIO (100 mg/kg of body weight) respectively for 12 weeks. Then, an 8-h fasting blood sample was collected for the detection of insulin. Results are expressed as the mean ± SD, n = 5. OXA: oxamate; PIO: pioglitazone. **: *p* < 0.01 as compared to the vehicle *db/+* mice; ##: *p* < 0.01 as compared to the vehicle *db/db* mice (one-way ANOVA followed by Student-Newman-Keuls test).

To observe the effects of OXA on insulin sensitivity in *db/db* mice, an ITT was conducted, and fasting blood insulin levels were determined after 12 weeks of treatment. As shown in Fig 6, treatment with 350, 550, and 750, but not 150, mg/kg of body weight of OXA improved insulin sensitivity in response to an ITT in *db/db* mice. Compared with the vehicle *db/db* mice, serum insulin levels of *db/db* mice administrated with OXA of 350, 550, and 750 mg/kg of body weight were decreased. The effect of OXA seemed to be dose-dependent; however, the differences of serum insulin levels were not statistically significant between the *db/db* mice receiving OXA at 350, 550, and 750 mg/kg of body weight (Fig 7).

### Effects of OXA on tissue lactate production and serum lactate levels

**Fig 8:**
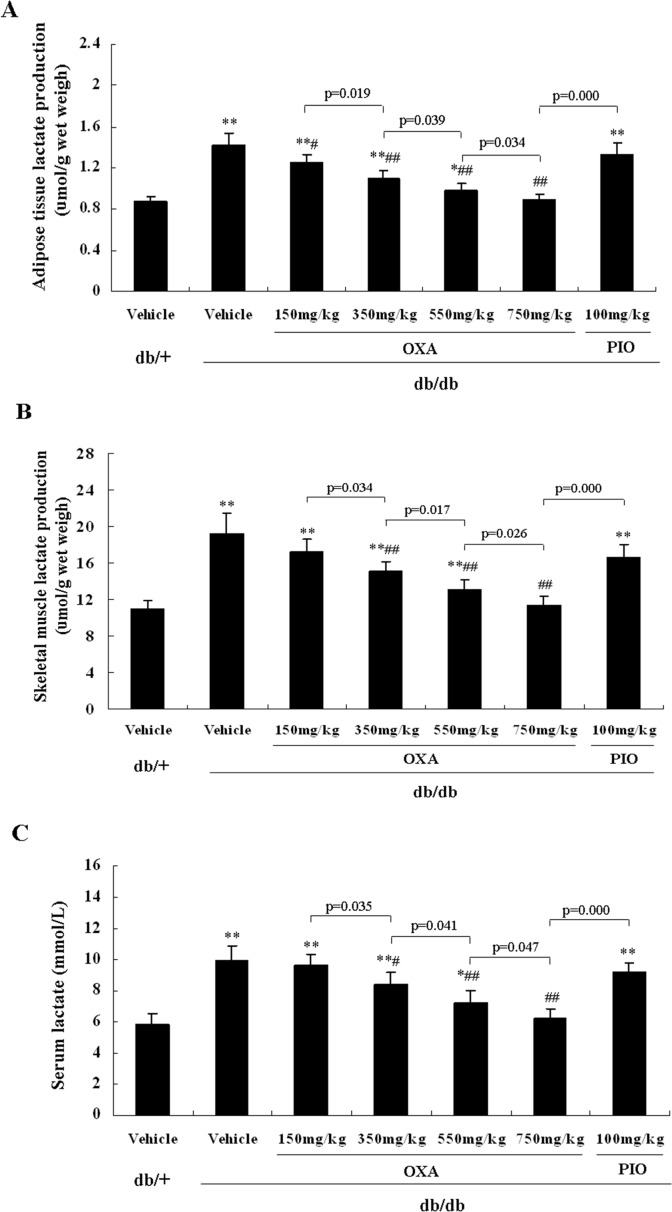
Effects of OXA on tissue lactate production and serum lactate levels. *db/+* mice received the vehicle (0.9% NaCl), and *db/db* mice received the vehicle and OXA (150, 350, 550, 750 mg/kg of body weight) or PIO (100 mg/kg of body weight) respectively for 12 weeks. Then an 8-h fasting blood sample was collected for the detection of lactate, and skeletal muscle and adipose tissue were dissected for the detection of lactate production. (A) Lactate production of adipose tissue; (B) lactate production of skeletal muscle; (C) serum lactate levels. Results are expressed as the mean ± SD, n = 5. OXA: oxamate; PIO: pioglitazone. *: *p* < 0.05 as compared to the vehicle *db/+* mice; **: *p* < 0.01 as compared to the vehicle *db/+* mice; #: *p* < 0.05 as compared to the vehicle *db/db* mice; ##: *p* < 0.01 as compared to the vehicle *db/db* mice (one-way ANOVA followed by Student-Newman-Keuls test).

After 12 weeks of treatment, the lactate production of adipose tissue and skeletal muscle and serum lactate levels were more increased in the vehicle *db/db* mice than those in the vehicle *db/+* mice. Treatment with 150 mg/kg of body weight of OXA decreased the lactate production of adipose tissue, but had no effect on the lactate production of skeletal muscle and serum lactate levels in *db/db* mice. However, administration of OXA at 350, 550, and 750 mg/kg of body weight decreased the lactate production of adipose tissue and skeletal muscle and serum lactate levels in *db/db* mice in a dose-dependent manner. Treatment with pioglitazone had not effect on the lactate production of adipose tissue and skeletal muscle and serum lactate levels in *db/d*b mice (Fig 8A–8C).

### Effects of OXA on serum levels of lipid and pro-inflammatory cytokines

**Fig 9:**
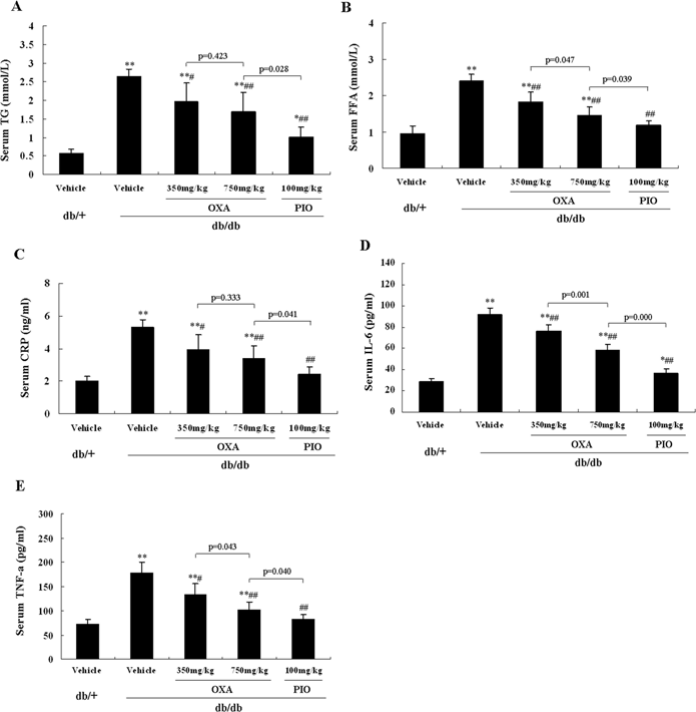
Effects of OXA on serum levels of lipid and pro-inflammatory cytokines. *db/+* mice received the vehicle (0.9% NaCl), and *db/db* mice received the vehicle and OXA (350 and 750 mg/kg of body weight) or PIO (100 mg/kg of body weight) respectively for 12 weeks. Then an 8-h fasting blood sample was collected for the detection of lipid and pro-inflammatory cytokines. (A) serum TG; (B) serum FFA; (C) serum CRP; (D) serum IL-6; (E) serum TNF-α. Results are expressed as the mean ± SD, n = 5. OXA: oxamate; PIO: pioglitazone. *: *p* < 0.05 as compared to the vehicle *db/+* mice; **: *p* < 0.01 as compared to the vehicle *db/+* mice; #: *p* < 0.05 as compared to the vehicle *db/db* mice; ##: *p* < 0.01 as compared to the vehicle *db/db* mice (one-way ANOVA followed by Student-Newman-Keuls test).

As shown in Fig 9, treatment with 350 and 750 mg/kg of body weight of OXA for 12 weeks decreased serum TG and FFA levels in *db/db* mice. The 750 mg/kg of body weight dose of OXA had more obvious effects on serum FFA (but not TG) levels than those of the 350 mg/kg of body weight dose of OXA (Fig 9A and 9B). Likewise, treatment with 350 and 750 mg/kg of body weight of OXA decreased serum CRP, IL-6, and TNF-α levels in *db/db* mice, and the 750 mg/kg of body weight dose of OXA had more obvious effects on serum IL-6 and TNF-α (but not CRP) levels than those of the 350 mg/kg of body weight dose of OXA in *db/db* mice (Fig 9C–9E).

### Effects of OXA on genes related to the insulin-signaling pathway in skeletal muscle

**Fig 10:**
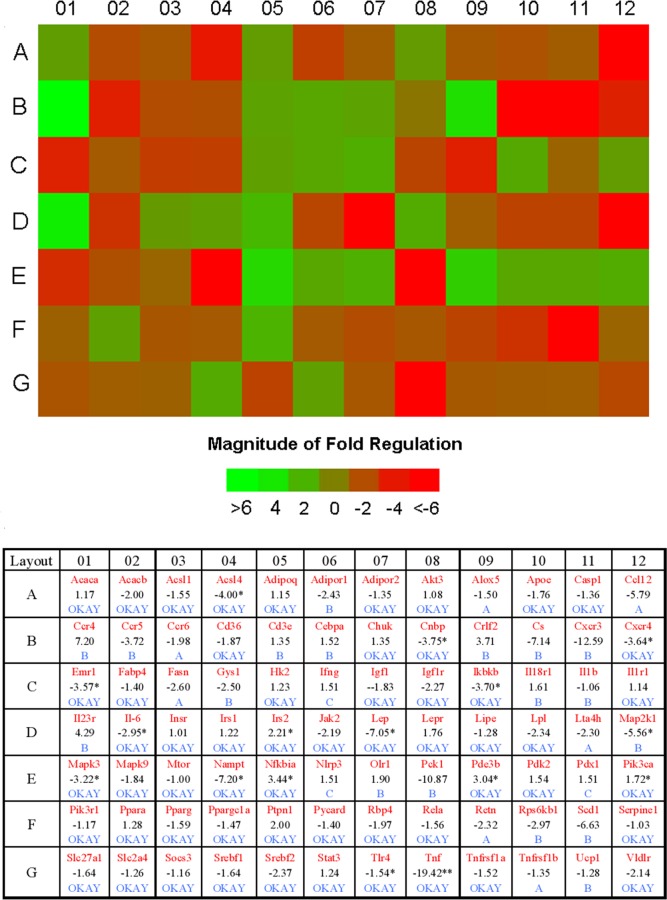
Effects of OXA on genes related to the insulin signaling pathway in skeletal muscle. *db/db* mice received the vehicle and OXA (750 mg/kg of body weight) for 12 weeks. Then, skeletal muscle was dissected.and the expressions of genes related to the insulin signaling pathway were measured by PCR array. Fold changes of the skeletal muscle genes in OXA-treated *db/db* mice compared with vehicle-treated *db/db* mice. *p < 0.05 and **p < 0.01 as compared to the vehicle-treated *db/db* mice (unpaired, two-tail Student’s *t*-test); (n = 3 for each group, the mRNA levels of all genes were normalized by β-actin). Abbreviated names for the genes are presented in red. Numbers (in black) represent fold-changes. A (in blue): This gene's average threshold cycle was > 30 in the control or the test sample, and was reasonably < 30 in the other sample. B (in blue): This gene's average threshold cycle was > 30, which means that the level of gene expression was relatively low in both samples, and the P-value for the fold-change was unavailable or high (*P* > 0.05). C (in blue): This gene's average threshold cycle was not determined or was > 35 (cut-off value) in both samples, indicating that gene expression was undetected, and the result was erroneous and non-interpretable. “OKAY” means that this gene's average threshold cycle was < 30 in both samples, and the result was accepted.

To compare the expression of genes related to the insulin response in OXA (750 mg/kg of body weight)-treated and vehicle-treated *db/db* mice, we used a PCR array for skeletal muscle. According to the criteria of at least a 2.0-fold-change in the experiment sets with statistically significant differences (*p* < 0.05), we found that the mRNA levels of acyl-CoA synthetase long-chain family member 4 (Acsl4), cellular nucleic acid binding protein (Cnbp), chemokine (C-X-C motif) receptor 4 (Cxcr4), EGF-like module containing, mucin-like, hormone receptor-like sequence 1 (Emr1), interleukin 6 (Il6), leptin (Lep), inhibitor of kappaB kinase beta (Ikbkb), nicotinamide phosphoribosyltransferase (Nampt), mitogen-activated protein kinase kinase 1 (Map2k1), Mitogen-activated protein kinase 3 (Mapk3), and tumor necrosis factor (Tnf) were decreased, while insulin receptor substrate 2 (Irs2), nuclear factor of kappa light polypeptide gene enhancer in B-cells inhibitor, alpha (Nfkbia) and Phosphodiesterase 3B, cGMP-inhibited (Pde3b) were increased in OXA-treated *db/db* mice (Fig 10).

### Effects of OXA on insulin secretion, islet morphology, and insulin expression

**Fig 11:**
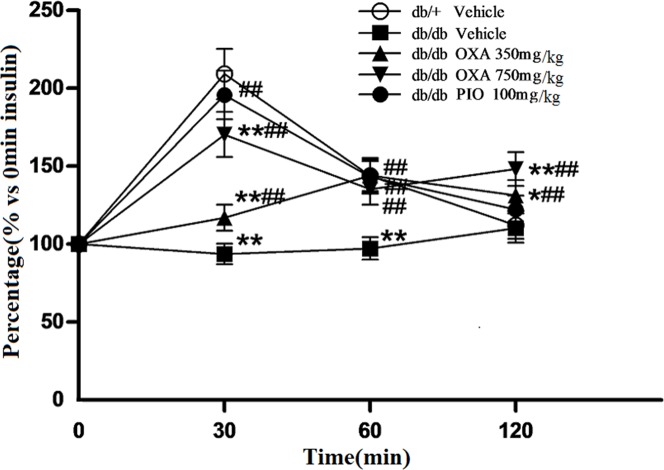
Effects of OXA on insulin secretion during IPGTT. *db/+* mice received the vehicle (0.9% NaCl), and *db/db* mice received the vehicle and OXA (350 and 750 mg/kg of body weight) or PIO (100 mg/kg of body weight) respectively for 12 weeks. After fasting for 10 h, the mice were subjected to a glucose tolerance test (GTT) by the intraperitoneal injection of 2 g/kg of body weight of glucose. Blood was sampled and examined at 0 (baseline), 30, 60, and 120 min for insulin. Results are expressed as the percentage (% versus 0 min insulin) ± SD, n = 5. OXA: oxamate; PIO: pioglitazone. *: *p* < 0.05 as compared to the vehicle *db/+* mice; **: *p* < 0.01 as compared to the vehicle *db/+* mice; ##: *p* < 0.01 as compared to the vehicle *db/db* mice (one-way ANOVA followed by Student-Newman-Keuls test).

In the vehicle *db/+* mice and PIO-treated *db/db* mice after 12 weeks of treatment, peak serum insulin levels occurred at 30 min during IPGTT, but no peak plasma insulin levels were observed in the vehicle *db/db* mice. Peak serum insulin levels appeared at 60 min in the *db/db* mice receiving OXA at 350 mg/kg of body weight, and at 30 min in the *db/db* mice receiving OXA at 750 mg/kg of body weight (Fig 11).

**Fig 12:**
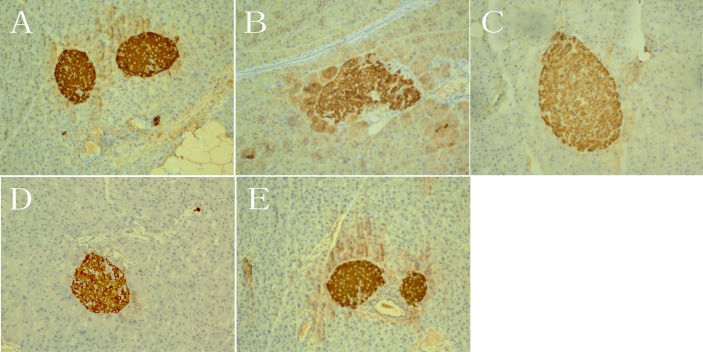
Effects of OXA on islet morphology and insulin expression. *db/+* mice received the vehicle (0.9% NaCl), and *db/db* mice received the vehicle and OXA (350 and 750 mg/kg of body weight) or PIO (100 mg/kg of body weight) respectively for 12 weeks. Then, the pancreas was dissected and insulin staining was performed (×200). (A) pancreas from vehicle *db/+* mice; (B) pancreas from vehicle *db/db* mice; (C) pancreas from *db/db* mice treated with OXA of 350 mg/kg of body weight; (D) pancreas from *db/db* mice treated with OXA of 750 mg/kg of body weight; (E) pancreas from *db/db* mice treated with PIO of 100 mg/kg of body weight. OXA: oxamate; PIO: pioglitazone.

After 12 weeks of treatment, the pancreatic islets of vehicle *db/+* mice and PIO-treated *db/db* mice maintained normal morphologies, and insulin staining was dark brown (Fig 12A and 12E). In contrast, some of the islets of the vehicle *db/db* mice became larger and irregular, some of the islets lost boundary definition, the arrangement of the cells in the islet became loose, and insulin staining was shallow (Fig 12B). Compared with the vehicle *db/db* mice, the morphology of the islet became regular, cells in the islet were relatively orderly arranged, and insulin staining was darker in the *db/db* mice receiving OXA at 350 and 750 mg/kg of body weight. Furthermore, the size of the islets became smaller and the insulin staining of cells became darker in the *db/db* mice receiving OXA at 750 mg/kg than those receiving 350 mg/kg of body weight (Fig 12C and 12D).

### Effects of OXA on LDH-A expression in islets

**Fig 13:**
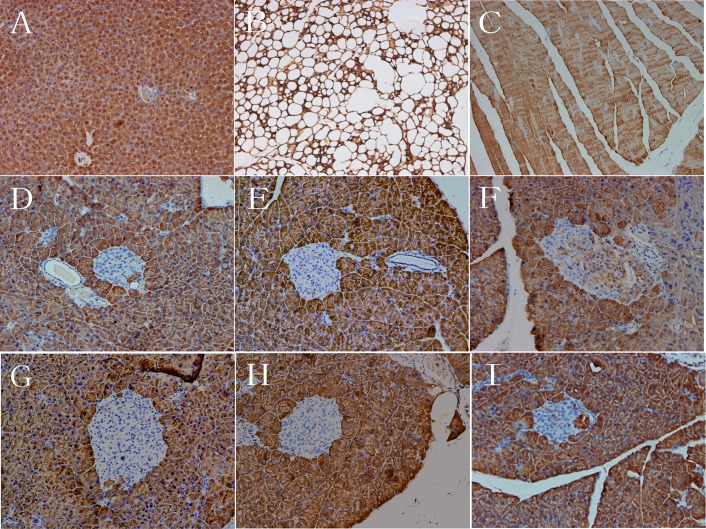
Effects of OXA on LDH-A expression in islets. Liver, adipose, skeletal muscle, and pancreas tissue from 4-week-old *db/+* mice, and pancreas tissue from the mice after a treatment (vehicle, OXA, and PIO) of 12 weeks were dissected and LDH-A staining was performed (×200). (A) Liver from 4-week-old *db/+* mice; (B) adipose tissue from 4-week-old *db/+* mice; (C) skeletal muscle from 4-week-old *db/+* mice; (D) pancreas from 4-week-old *db/+* mice; (E) pancreas from vehicle *db/+* mice; (F) pancreas from vehicle *db/db* mice; (G) pancreas from *db/db* mice treated with OXA of 350 mg/kg of body weight; (H) pancreas from *db/db* mice treated with OXA of 750 mg/kg of body weight; (I) pancreas from *db/db* mice treated with PIO of 100 mg/kg of body weight. OXA: oxamate; PIO: pioglitazone.

To observe LDH-A expression in the tissue of normal mice, and to investigate the effects of OXA on LDH-A expression in the islets of *db/db* mice, liver, adipose, skeletal muscle, and pancreas tissue from 4-week-old *db/+* mice and pancreas tissue from 16-week-old (treated for 12 weeks) treated *db/+* and *db/db* mice were dissected and were stained with LDH-A. In the 4-week-old *db/+* mice, almost all of the cells in the liver (Fig 13A), adipose (Fig 13B), skeletal muscle (Fig 13C), and pancreatic exocrine tissue were stained positively for LDH-A, however, the cells in the islets were hardly stained for LDH-A (Fig 13D). Likewise, almost all of the cells in pancreatic exocrine tissue were stained positively for LDH-A in the 16-week-old vehicle *db/+* mice and all treated *db/db* mice (Fig 13E–13I). LDH-A positive cells in islets were found predominantly in the vehicle *db/db* mice, while only a few cells in islets were lightly stained for LDH-A in the vehicle *db/+* mice, the *db/db* mice receiving OXA at 350 and 750 mg/kg of body weight, and PIO at 16 weeks of age (Fig 13E–13I).

## Discussion

In the present study, we evaluated the effects of OXA on glycemic control and insulin sensitivity in *db/db* mice. Our results show that a treatment of OXA (350–750 mg/kg body weight) for 12 weeks significantly decreased the fasting and post-load blood glucose and HbA1c levels in the *db/db* mice. At the same time, OXA also improved insulin secretion and the morphology of pancreatic islets in the *db/db* mice. Meanwhile, OXA-treated mice were significantly more responsive to insulin and the blood insulin level was significantly decreased, indicating that the treatment of OXA ameliorated insulin resistance in *db/db* mice. These effects of OXA in the *db/db* mice were similar to that of pioglitazone; the difference was that treatment of OXA decreased the body weight, whereas pioglitazone significantly increased the body weight gain of the *db/db* mice.

OXA is a pyruvate-competitive inhibitor of LDH, which catalyzes the conversion process of pyruvate into lactate. In the fasted, resting state, lactate is produced by skeletal muscle, adipose tissue, the brain, erythrocytes, the liver, the gut, the kidneys, and skin, and both skeletal muscle and adipose tissue account for the majority of the lactate released [14]. Elevated blood lactate is a marker for cellular stress [30]. On the other hand, it was also demonstrated that lactate induced insulin resistance, and lactate treatment inhibited insulin action by inhibiting the insulin receptor substrate-1 (IRS-1) and IRS-2 mediated PI3K and PKB pathways [33, 34]. In animal models of type 2 diabetes, increased blood lactate levels and the production of lactate in adipose tissue and skeletal muscle had been observed [33, 35–37]. Earlier and recent studies, including retrospective and prospective studies, have suggested that blood lactate was strongly associated with insulin resistance and type 2 diabetes and was an independent risk factor of diabetes development [8–11, 14, 15]. In our study, the production of lactate was increased in adipose tissue and skeletal muscle, and serum lactate levels were elevated in vehicle *db/db* mice compared with vehicle *db/+* mice at 16 weeks of age. After the treatment of OXA for 12 weeks, the production of lactate in adipose tissue and skeletal muscle and serum lactate levels were decreased, accompanied by improvement of insulin resistance and glycemic control in *db/db* mice. Meanwhile, a PCR array of skeletal muscle showed that OXA upregulated (Irs2, Nfkbia, and Pde3b) or downregulated (Map2k1, Mapk3, Ikbkb, etc.) the expression of genes related to the insulin-signaling pathway. However, the treatment of pioglitazone had no effects on the production of lactate in adipose tissue and skeletal muscle and serum lactate levels, although it significantly improved insulin resistance and glycemic control. These results indicate that the increased lactate production of adipose tissue and skeletal muscle may be at least partially responsible for insulin resistance and diabetes in *db/db* mice, and the anti-diabetic roles of OXA were primarily mediated by the inhibition of the lactate production of adipose tissue and skeletal muscle, and thus the improvement of insulin sensitivity.

In the beta cell, a small group of genes, which are almost ubiquitously expressed in mammalian tissues, are specifically repressed, in order to prevent inappropriate release of insulin. Abnormal upregulation of these genes might lead to the deterioration of beta-cell function. This group of genes is recently referred to as ‘disallowed beta-cell genes’ [16, 17]. LDH-A is one of the 'founder' members of this group [16]. In vivo and in vitro studies have shown that high glucose levels upregulate the expression of LDH-A mRNA in rodent and human pancreatic islets [18–20]. In the present study, we observed that LDH-A was highly expressed in the liver, adipose tissue, skeletal muscle, and pancreatic exocrine tissue, but was hardly expressed in islets from 4-week-old *db/+* mice. However, LDH-A was prominently expressed higher in islets from the vehicle *db/db* mice than those from the vehicle *db/+* mice at 16 weeks of age. Our results, for the first time at the protein level, strongly support the concept of ‘disallowed beta-cell genes’. Interestingly, both the treatment of OXA and pioglitazone for 12 weeks significantly downregulated the expression of LDH-A in islets from *db/db* mice. These results indicate an improvement in insulin resistance and glycemic control, lowered LDH-A expression in islets (probably due to restructuring of the morphology of the pancreatic islets and an increased supply of blood and oxygen). Decreased LDH-A expression in islets might be related to an improvement of insulin secretion in *db/db* mice. Since LDH-A expression was prominently decreased in the islets of *db/db* mice with the treatment of OXA, the direct effects of OXA on the inhibition of the LDH activity of beta cells to improve insulin secretion might be inconspicuous.

Lipotoxicity constitutes an important pathogenic link between obesity, insulin resistance, and type 2 diabetes [24]. Elevated FFA impairs glucose oxidation/glycogen synthesis and decreases glucose transport/phosphorylation via activation of the NF-κB, and inhibition of the IRS-1, signaling pathways [25, 38]. On the other hand, some evidence has suggested a state of chronic low-grade inflammation is involved in the development of insulin resistance and the pathogenesis of type 2 diabetes [39]. It has been demonstrated that pro-inflammatory cytokines like TNF-α and IL-6 are able to interfere with the insulin-signaling pathway and decrease insulin action [40–42]. In the adipose tissue of obese or diabetic rats or humans, up to 50–70% of the glucose taken up is converted to lactate [35]. Thus, the adipose tissue macrophages in patients with obesity or diabetes are likely to be exposed to a high level of lactate [30]. It was observed that lactate boosted the gene transcription of pro-inflammatory cytokines in macrophages, and OXA inhibited lactate output, FFA de novo synthesis, and TG accumulation in 3T3-L1 cells [30, 31]. A recent study also showed that palmitate caused a striking loss of PKB phosphorylation in response to insulin and markedly stimulated NF-κB-mediated pro-inflammatory signaling in myotubes, and both of these effects were antagonized by OXA [32]. In the present study, a treatment of OXA for 12 weeks decreased the serum levels of TG, FFA, CRP, IL-6, and TNF-α in *db/db* mice. OXA also downregulated the expression of inflammation-related genes, such as Tnf, Il6, Emr1, and Cxcr4, which might be mediated by the inhibition of the MAPK/NF-κB pathway (based on the downregulation of Map2k1, Mapk3, and Ikbkb and the upregulation of Nfkbia) in the skeletal muscle of *db/db* mice. These results indicated that the improvement of insulin resistance by OXA might involve the reduction of lipotoxicity and inflammation in *db/db* mice.

We also found that treatment with OXA (350–750 mg/kg body weight) decreased body weight gain in db/db mice. However, the mechanism was unclear. Decreased food intake might not be a major contributor, since only the high dose of OXA (750 mg/kg body weight) lightly decreased food intake in db/db mice. An in vitro study of 3T3-L1 cells showed that OXA inhibited FFA synthesis and TG accumulation [31], but the effects of OXA on fat accumulation in vivo in db/db mice need to be further studied.

Although OXA treatment improved insulin resistance and glycemic control, it failed to prevent the development of diabetes in db/db mice. The db/db mice were treated with OXA at the age of 4 weeks, when blood glucose levels were normal. However, all the db/db mice became diabetic (FBG ≥ 13.9 mmol/L) at the age of 6 weeks. OXA began to take effect on glycemic control after 5–7 weeks of treatment. One possible explanation for this phenomenon was that elevated blood glucose, together with FFA, significantly increased lactate production in the tissues of db/db mice at about the age of 10 weeks, which in return exacerbated insulin resistance and hyperglycemia. However, at this time point, OXA began to exert effects on glycemic control.

It is worth pointing out that in addition to the inhibition of LDH, OXA also inhibits pyruvate carboxylase (PC) and aspartate aminotransferase (AAT) [43, 44], which might have some effects on the glucose metabolism of cells of the liver, adipose tissue, and skeletal muscle. PC is a member of the biotin-dependent enzyme family and catalyzes the ATP-dependent carboxylation of pyruvate to oxaloacetate [45]. In mammals, PC plays a crucial role in gluconeogenesis and lipogenesis [45]. Increased expression of PC has been reported in obese Zucker Diabetic Fatty (ZDF) rats, and targeting the inhibition of PC expression in liver and adipose tissue reduces gluconeogenesis and adiposity and improves insulin resistance [46]. Earlier in vitro studies suggest that OXA inhibits hepatic gluconeogenesis via non-competitive inhibition of PC and the competitive inhibition of the mitochondrial pyruvate translocator protein [43, 47, 48]. AAT is an ubiquitous pyridoxal phosphate-dependent enzyme, and functions together with malate dehydrogenase to form the NADH shuttle system, which plays an essential role in the coupling of glycolytic metabolism and mitochondrial generation of energy [44]. Accordingly, the inhibition of OXA on AAT might have deleterious effects on the glucose metabolism of cells of the liver, skeletal muscle, and adipose tissue. Therefore, the effects of the inhibition of PC and AAT by OXA in vivo on insulin resistance and glycemic control in *db/db* mice should be further investigated.

Aerobic glycolysis is a hallmark of malignant tumor metabolism, and OXA can inhibit the activity of LDH and decrease lactate production in cancer cells [2, 3]. It has been reported that OXA has anti-cancer properties in vitro and in animals in vivo [1, 5, 6, 49, 50]. However, OXA is a competitive inhibitor of LDH at high concentrations, which limits its therapeutic potential in clinical practice [49]. Oxamic acid derivatives are developing, which are being considered as a potential drug for the treatment of malignant tumors in the future [7, 51]. In the present study, we demonstrated that OXA improved glycemic control and insulin sensitivity in *db/db* mice. Therefore, we consider that Oxamic acid derivatives may also be a potential drug for the treatment of type 2 diabetes.

In summary, our results show that increased lactate production of adipose tissue and skeletal muscle may be at least partially responsible for insulin resistance and diabetes in *db/db* mice. Treatment with OXA (350–750 mg/kg of body weight) improved glycemic control and insulin sensitivity in *db/db* mice primarily via the inhibition of tissue lactate production, and the reduction of lipotoxicity and inflammation. Oxamic acid derivatives may be a potential drug for the treatment of type 2 diabetes.
